# Spiders in space—orb-web-related behaviour in zero gravity

**DOI:** 10.1007/s00114-020-01708-8

**Published:** 2020-12-03

**Authors:** Samuel Zschokke, Stefanie Countryman, Paula E. Cushing

**Affiliations:** 1 Section of Conservation Biology (NLU), Department of Integrative Biology, University of Basel, 4056 Basel, Switzerland; 2 BioServe Space Technologies, University of Colorado, Boulder, CO 80309-0429 USA; 3 Denver Museum of Nature & Science, Denver, CO 80205 USA

**Keywords:** Extended phenotype, Golden silk orb weaver, Microgravity, *Nephila*, Sensory compensation, Spider web

## Abstract

Gravity is very important for many organisms, including web-building spiders. Probably the best approach to study the relevance of gravity on organisms is to bring them to the International Space Station. Here, we describe the results of such an experiment where two juvenile *Trichonephila clavipes* (L.) (Araneae, Nephilidae) spiders were observed over a 2-month period in zero gravity and two control spiders under otherwise identical conditions on Earth. During that time, the spiders and their webs were photographed every 5 min. Under natural conditions, *Trichonephila* spiders build asymmetric webs with the hub near the upper edge of the web, and they always orient themselves downwards when sitting on the hub whilst waiting for prey. As these asymmetries are considered to be linked to gravity, we expected the spiders experiencing no gravity to build symmetric webs and to show a random orientation when sitting on the hub. We found that most, but not all, webs built in zero gravity were indeed quite symmetric. Closer analysis revealed that webs built when the lights were on were more asymmetric (with the hub near the lights) than webs built when the lights were off. In addition, spiders showed a random orientation when the lights were off but faced away from the lights when they were on. We conclude that in the absence of gravity, the direction of light can serve as an orientation guide for spiders during web building and when waiting for prey on the hub.

## Introduction

### General background

Gravity influences many organisms in a wide variety of ways. Among many others, gravity induces the directed growth of roots and stem in plants (Chen et al. 1999), it is an important cue for honey bees whilst dancing on the honeycomb (von Frisch 1967), and it causes elephants to walk around hills rather than across them (Wall et al. 2006). Gravity is probably the reason for the sexual size dimorphism in some spider species (Moya-Laraño and Foellmer 2016), and last but not least, gravity influences the prey capture behaviour of spiders building vertical orb webs (ap Rhisiart and Vollrath 1994; Herberstein and Heiling 1999; Coslovsky and Zschokke 2009), which in turn affects the structure of these orb webs, resulting in vertical asymmetries in many different ways (Mayer 1952; Eberhard 2014; Zschokke and Nakata 2015). Probably the most obvious asymmetry in orb webs is the hub position: in most orb webs, the hub is positioned in such a way that the capture area below the hub is larger than the capture area above the hub (e.g. Mayer 1952; Witt and Reed 1965; ap Rhisiart and Vollrath 1994). Empirical and theoretical studies suggest that this asymmetry is mainly an adaptation to the spider’s prey capture behaviour and that it reflects the spider’s ability to run downwards faster than upwards (Masters and Moffat 1983; ap Rhisiart and Vollrath 1994; Maciejewski 2010; Zschokke and Nakata 2010). Another striking vertical asymmetry is the orientation of the spider whilst waiting on the hub for prey to be intercepted by the web: with very few exceptions, spiders face downwards when waiting on the hub, which is also considered to be an adaptation to prey capture behaviour (Maciejewski 2010; Nakata and Zschokke 2010; Zschokke and Nakata 2010).

As outlined above, gravity does play an important role in prey capture and web structure, and therefore, we can expect that gravity is also important during web building. However, to really understand the influence of gravity on web building, it is necessary to conduct experiments in which the forces acting roughly parallel to the web plane (i.e. in the same direction as gravitational forces act for vertical webs) are increased or decreased during web building.

### Previous experiments and observations on web building under altered gravity

One approach to *increase* the force acting on the spider is to glue small weights onto its abdomen (Mayer 1952; Vollrath and Köhler 1996; Herberstein and Heiling 1999; Coslovsky and Zschokke 2009). In these cited studies, the influence of the added weight on the vertical position of the hub was analysed. Interestingly, however, their results differed: Herberstein and Heiling (1999), who had assessed the first web built after adding the weight, found an increased asymmetry in the webs built by the heavier spiders, whereas Vollrath and Köhler (1996), who analysed the second web after adding the weight, as well as Coslovsky and Zschokke (2009), who assessed webs built 1 week after adding the weight, did not find any difference in vertical web asymmetry between experimental and control spiders.

Another approach to increase the force acting on the spider is to put the spiders in a centrifuge during web building. The authors of such a study reported no obvious change in geometry for webs built under 3.5 g, but reported that “the geometry of webs built at 15 g […] was significantly different from that of orb webs built under our standard laboratory conditions” (Vollrath and Köhler 1996, p. 388), but the authors did not provide any details of the observed differences.

Whilst increasing forces acting parallel to gravity is relatively easy, it is much more difficult to reduce or even eliminate gravity. Nevertheless, there are approaches that eliminate *constant* forces acting parallel to the web, i.e. in the direction gravity acts on the spider in vertical orb webs. One such approach is to induce the spider to build a horizontal web. In *Argiope argentata* (Fabricius), the number of spiral turns above and below the hub was more even when spiders were forced to build the web in a horizontal rather than in a vertical position (Nogueira and Ades 2012). Unfortunately, most other spiders that usually build vertical webs refuse to start building a web when the space offered allows only horizontal webs. However, since at least some spiders are willing to continue building a web horizontally, once they have started building a vertical web, it is possible in laboratory experiments to lay the web horizontally at a certain stage during web building, e.g. after auxiliary spiral completion (Vollrath 1986). The spirals of experimental webs built by *Araneus diadematus* (L.) in such a way were rounder and had a reduced vertical asymmetry, indicating that the spiders were missing gravity as a cue to build webs with their typical elongated and asymmetric spirals (Mayer 1952; Zschokke 1993, 2011). Similarly, it was found in spiders which usually build slanted webs, that the sticky spiral asymmetry increased with the angle of the web to the horizontal; i.e. more vertical webs had more asymmetric sticky spirals (Eberhard 1987; Gregorič et al. 2013; Tew and Hesselberg 2018).

Rotating webs around an axis perpendicular to the web plane during web building is another approach to study web building without a constant gravity acting parallel to the web surface. This can be done either by continuous rotation with a certain rotational speed, as in a clinostat, or by quickly rotating the web by 90° or 180° at a specific stage of web building. Continuous rotation leads to more or less severe distortions of the completed web, depending on rotational speed (Mayer 1952; Vollrath 1986, 1988). In webs that were quickly rotated at a specific stage of building, some aspects of the web built after rotation followed the original web orientation, whereas others followed the gravity vector after web rotation (Peters 1937a; König 1951).

All these observations and experiments strongly suggest that gravity is an important factor during web building, but they cannot answer the question, whether spiders can build webs in zero gravity and, if yes, how the zero-gravity environment will affect the completed web. These questions can only be answered by bringing spiders into a zero-gravity environment, i.e. by bringing spiders into space.

### Observations from previous experiments on web-building spiders in zero gravity

The first time spiders were brought into space was in July 1973, when two *A. diadematus* spiders were brought to Skylab to observe their web building. This experiment had been suggested by Judith Miles, a high school student, in the NASA Skylab Student Experiment Competition. Both spiders built several webs, but the web structure could not be comprehensively evaluated, since there were no photographs showing the entire web. Only five webs were photographed, and only one of them, the second one built by one spider, was apparently regular, whereas the other four “were of small size and highly irregular spacing” (Witt et al. 1977, p. 117). Furthermore, the later webs were reported to be “highly irregular” (Witt et al. 1977, p. 121). In contrast to webs built by *A. diadematus* in normal gravity, which have smaller angles between radii below than above the hub (Peters 1937b; Mayer 1952), the one “regular web” built in Skylab showed no apparent asymmetry in angles between radii, and there seemed to be fewer U-turns in the sticky spiral compared to webs built in normal gravity (Witt et al. 1977). However, the lack of provisions of moisture or food for the experimental spiders made it difficult to determine if the space web irregularity was due to changes in gravity or changes in the spider’s condition. Nevertheless, this experiment clearly demonstrated that *A. diadematus* are able to build webs even in the absence of gravity.

In 2008, spiders were again brought into space to build orb webs. But this time, photographing the resultant webs was better planned and the spider husbandry was much improved by bringing along *Drosophila* colonies to provide a continuous food supply for the spiders. Since the results of this experiment have not been formally published, we take the opportunity here to briefly report on it, even though it is not the main focus of this paper. In this experiment, two different orb-web spiders, an adult female of the small orb-weaving species, *Metepeira labyrinthea* (Hentz) wild caught in FL, USA, by Mark Stowe, and a juvenile *Larinioides patagiatus* (Clerck) lab-reared and collected from a population in CO, USA, were brought to the International Space Station (ISS). *M. labyrinthea* was put into the main observation chamber of the spider habitat of which the front-facing side was optically clear plastic allowing for imaging of the spider’s movements and web-building behaviours, and *L. patagiatus* was put into a small holding chamber in the same habitat with the intention to release it into the main observation chamber should *M. labyrinthea* die soon after arrival to the ISS. However, the backup spider *L. patagiatus* somehow managed to escape from its holding chamber into the main observation chamber leading to the situation that both spiders were in the main observation chamber together. The lights in the habitat were turned on for 12 h every day, and the spider behaviour was recorded by taking a picture of the main observation chamber every 5 min and 17 s. In addition, some sequences were recorded on video.

**Fig. 1:**
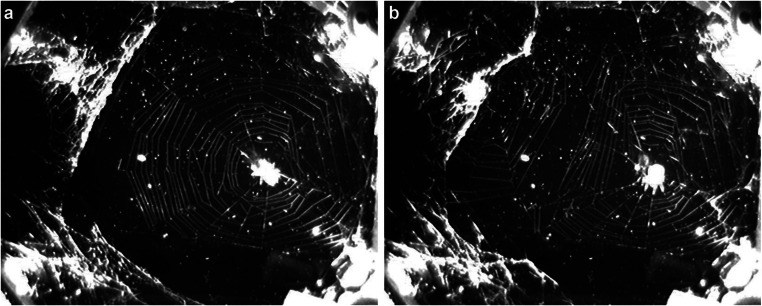
In 2008, orb-web spiders were brought for the first time to the ISS, where they built some webs in zero gravity. Unfortunately, both spiders were accidentally released into the same habitat and therefore interfered with each other when building their webs. **a** First web of *Larinioides patagiatus* built in the ISS. **b** View of the habitat after the second spider, *Metepeira labyrinthea*, had completed its web, which caused a partial destruction of the first *L. patagiatus* web

In the first days after launch, the spiders moved around in the observation chamber resulting in it being filled with seemingly random silk strands. On the 6th day after launch, on 20 November 2008, *L. patagiatus* built a web (Fig. 1a), and about 1 h later, *M. labyrinthea* also built its first web, which caused a partial destruction of the *L. patagiatus* web (Fig. 1b). Neither of these first webs was very regular, but they were clearly recognisable and functional as orb webs. Eight hours later, just when the lights were turned off, *L. patagiatus* started building its second web, which it removed shortly before the lights were turned on again, making it therefore impossible to evaluate its structure. A few hours later, *M. labyrinthea* built its second web, which was again not very regular. Altogether six more webs could be recorded during the following eight days, all much less regular than the first ones.

Since the design of the food cup that held the fruit fly larvae and eggs below the observation chamber allowed for unlimited access of the fruit fly larvae to the chamber, and because the fruit fly larvae population increased more than expected, the fruit fly larvae and pupae started covering up the observation window about 2 weeks after the launch. After approximately 1 month, they had completely covered the viewing window, making it impossible to see the spiders and their movements within the observation chamber.

The available observations of the spiders and their webs have clearly shown that the spiders used in this experiment were able to build functional orb webs in zero gravity. Unfortunately, it turned out to be more difficult than expected to determine whether shape and structure of the webs built in zero gravity differed from the control webs. This was due to the irregularity of the webs built in zero gravity, the small sample size of experimental webs, the difficulty to distinguish the two spiders with certainty based on the low-resolution pictures, and the small expected differences between the only slightly asymmetric control webs of *L. patagiatus* and its webs built in zero gravity.

### Introduction to experiment with *Trichonephila* spiders

When the opportunity arose to do another experiment in 2011, we decided—based on the above conclusions from the 2008 experiment—to use spiders which build highly asymmetric webs under normal gravitational conditions in order to increase the probability to be able to detect a difference in web shape between webs built in zero gravity and the control webs. In addition, we could also increase the independent sample size by using two habitats simultaneously, each loaded with the same species. To eliminate the previous issue with the fruit fly larvae on the viewing window of the habitat, the fruit fly habitat area was also reconfigured, thus extending the observation period to 2 months.

**Fig. 2:**
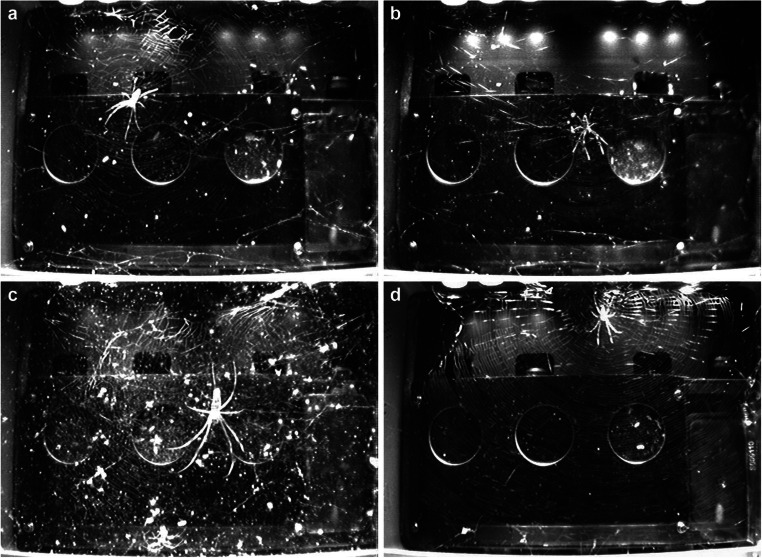
In 2011, two *Trichonephila clavipes* spiders were brought to the ISS in separate habitats, where their web building could be observed for almost 2 months under zero-gravity conditions. At the same time, two spiders were kept in identical habitats on the ground. **a** Symmetric web built in zero gravity (7^th^ web of spider B, asymmetry = −0.23, regularity = 3.9). **b** Asymmetric web built in zero gravity (5^th^ web of spider B, asymmetry = −0.53, regularity = 7.4). **c** Web built after 23 days in zero gravity (19^th^ web of spider B, asymmetry = −0.16, regularity = 1.3); this web was quite chaotic (which was typical for later webs built in zero gravity) and not well visible due to the build-up of dirt on front glass and rear of habitat. **d** Web of control spider built in normal gravity (4^th^ web of spider D, asymmetry = −0.83, regularity = 7.5). For descriptions on how asymmetry and regularity were assessed, see text

For the 2011 experiment, which is the main focus of the present study, we chose *Trichonephila clavipes* (L.) (previously known as *Nephila clavipes*; Kuntner et al. 2018), commonly known as golden silk orb weaver, since their webs are almost always highly asymmetrical (only the very first webs built by newly emerged spiderlings are sometimes vertically symmetric; Hesselberg 2010; W.G. Eberhard, pers. comm.). Another advantage of *T. clavipes* is that—thanks to their much-elongated abdomen—their orientation on the hub is clearly recognisable, even in low-quality pictures (cf. Fig. 2).

Since the vertical asymmetry of webs, as well as the spider orientation, are generally considered to be linked to gravity, we expected our *T. clavipes* spiders experiencing no gravity to either place the hub near a random edge of the web, and to orient themselves towards the larger part of the web when sitting on the hub, or to build webs with the hub in the centre (as 1st instar spiderlings of many other *Nephila* species do; Bleher 2000; Shinkai 1985) and to show an inconsistent or random orientation when sitting on the hub.

## Material and methods

“Spiders in Space” was a K-12 educational experiment whose scientific objective was to “examine orb-web building behaviour over a 45-day period” (Bhattacharya et al. 2019). For this experiment, *T. clavipes* egg sacs were collected on the grounds of Kennedy Space Center in Cape Canaveral Florida and transferred to the Butterfly Pavilion, Westminster, CO, where the spiderlings hatched and were kept until they were needed for the experiment. Four of those then juvenile *T. clavipes* were selected, with the aim to choose females (as it turned out, two were males after all, cf. Table 1). Two of those spiders were brought to the ISS where they were exposed to a zero-gravity environment, and the other two, serving as controls, were kept at the BioServe Space Technologies’ labs located within the University of Colorado Boulder under conditions, which were identical to those in the ISS except for gravity.

**Table 1:** Overview of the four spiders used in the experiments. Spider A (named “Gladys” by the astronauts) was still alive when it was returned to Earth with the final Space Shuttle mission, whereas spider B (“Esmeralda”) died in space. In the column “# webs”, the first number indicates the total number of webs built, and numbers in parentheses indicate the number of webs for which the vertical asymmetry could be assessed, and for which the regularity could be assessed, respectively

ID	Gravity	Sex	# webs	# moults	1st web	Last web	Death
A	Zero	M	22 (15, 17)	2	20 May	16 June	> 21 July
B	Zero	F	34 (16, 30)	3	19 May	29 June	15 July
C	Normal	M	17 (14, 16)	1	20 May	17 June	> 12 July
D	Normal	F	33 (17, 31)	3	20 May	7 July	> 12 July

During the experiment, all four spiders were kept singly in cubic habitats (17.3 cm wide × 12 cm tall × 5.5 cm deep), which were lit from one side by six white LED lamps for 12 h, followed by 12 h of darkness. As the lights were placed at the top of the control habitats, we always refer to the side with the lights as “top”, also in the zero-gravity treatments. During the dark period, one habitat in zero gravity and one habitat in normal gravity were lit by infrared LEDs, allowing us to record images around the clock (it had been intended to use infrared lighting in all habitats, but because of overheating in the camera system, the infrared lighting had to be turned off in one of the two habitats both on the ground and in zero gravity). Humidity levels matched the ambient humidity levels onboard the International Space Station, which is typically 50%. Both the flight and ground habitats were placed inside one BioServe incubator called Commercial Generic Bioprocessing Apparatus (CGBA) which controlled the habitat temperature to 25 °C. The spider habitats were custom built to fit two into one CGBA. The habitats with the spiders were launched aboard Space Shuttle Endeavour on May 16, 2011, and transferred to the International Space Station on May 19, 2011. In order to keep the spiders from building webs prior to being transferred into CGBA on orbit, the spiders were housed in a small compartment within the spider habitat which was large enough for the spider to move but not large enough to build a web. Water via a wicking system was also provided to the spider in this compartment. The spiders were released from this small compartment upon installation into CGBA on May 19, 2011.

*Drosophila* fruit flies were provided from a custom-designed habitat that was secured to the back inside wall of the spider habitats. The *Drosophila* habitat had three separate compartments containing *Drosophila* medium supplemented with ground dog food for added protein (Mayntz et al. 2009). One compartment was seeded with eggs, larvae and pupae 7 days prior to launch. Upon installation of the habitats into CGBA, the seeded compartment was opened and then closed by the crew after the release of about 10 newly hatched flies. The second unseeded compartment was opened at the same time and remained open until the next feeding session in order for the released flies to access this fresh food and seed this compartment. This was repeated for the third fresh food compartment at a later feeding enabling the extended culture of the fruit fly colony. Flies were released into the spider compartment at 6 different times during the experiment with the last one occurring July 8, 2011.

All activities of each spider were recorded by taking pictures with 3 cameras per spider, one camera covering c. 90% of the entire habitat, one camera covering a limited part of the habitat on the right-hand side and one camera with a close-up view of an area on the left-hand side. Each camera took pictures at a resolution of 640 × 480 pixels at regular intervals of mostly 5 min and 17 s. During some periods, especially during the first days, the intervals were longer than 5 min, and there were some gaps in the recording due to technical problems. The recordings ended on 18 July (i.e. after 60 days) for the zero-gravity treatment and on 12 July (i.e. after 54 days) for the controls. In total, about 130,000 pictures were taken.

### Web asymmetry

The vertical asymmetry of the webs was measured in the pictures showing the entire habitat by determining the vertical hub position, as well as the uppermost and lowermost part of the sticky spiral (Kuntner et al. 2010). When the uppermost or lowermost turn of the sticky spiral were not well visible in the picture of the completed web, their position was determined by comparing the last picture taken before those threads were built with the first picture taken after these threads were built, which helped to better distinguish the relevant threads. In those cases, where the threads were still not discernible (in 7 of the 63 webs, some threads were just beyond the edge of the picture), their position was estimated based on the visible part of the sticky spiral. Following Zschokke (1993) and Hesselberg (2010), the asymmetry was calculated as (*upper* − *lower*)/(*upper* + *lower*), where *upper* was the vertical distance between the centre of the hub and the uppermost part of the sticky spiral, and *lower* was the vertical distance between the centre of the hub and the lowermost part of the sticky spiral. Symmetric webs thus had an asymmetry of 0, and webs in which the hub was above the geometric centre had negative asymmetry values. Due to the continuous build-up of debris and unused spider silk which obstructed the pictures too much to assess the position of threads with reasonable certainty, web asymmetry could not be assessed after about 20 webs had been built (Fig. 2c). We compared web asymmetries using Mann-Whitney *U* tests and validated their results with a general linearised mixed model (glmmPQL) with spider ID as a random factor.

### Spider orientation

The spider orientation was assessed in all pictures taken after it had built the sticky spiral at least partially (the spider sometimes interrupted sticky spiral building to retrieve prey caught in the web) and in which the spider was on the hub in its typical resting position with all legs extended (cf. Fig. 2). In most cases, the spiders removed the web within less than 24 h after sticky spiral building, but for webs which were left intact for more than 24 h, we only evaluated pictures taken during the first 24 h. In each picture, the angle was categorised in 5° steps, i.e. spiders facing vertically downwards were considered to have an orientation of 177.5° (i.e. the average between 175 and 180°). In total, we assessed the spider orientation in 100 webs based on 14,528 pictures, of which 14,021 showed the spider in its resting position and could therefore be used for the analysis.

### Web regularity

To assess web regularity, we evaluated sticky spiral evenness, sticky spiral concentricity and the presence of gaps in the sticky spiral where the auxiliary spiral is (cf. Fig. 2d). Due to the subjectivity of this approach, each web was scored eight times. In each scoring, the webs were presented in random order in such a way that the evaluator (SZ, SC and PEC) neither knew whether the web was built in zero gravity or in normal gravity nor when the web was built. The sticky spiral evenness was scored on a scale from 0 (irregular spacing between subsequent sticky spiral loops, threads not parallel) to 4 (very even spacing between subsequent sticky spiral loops (except for the gaps, see below) and the sticky spiral threads are parallel to each other). The sticky spiral concentricity was scored on a scale from 0 (subsequent sticky spirals cross each other) to 2 (the centre of all sticky spirals is at the same place). The presence of gaps in the sticky spiral where the auxiliary spiral is was also scored on a scale from 0 (no gaps observable) to 2 (regular gaps in the sticky spiral in large parts of the web). The three scores were added up and averaged across the eight assessments to give an overall assessment of web regularity. We could score the regularity of 47 webs built in zero gravity and for 47 webs built in normal gravity (cf. Table 1). To assess the influence of gravity and of the day on web regularity, we used a general linearised mixed model (glmmPQL) with the factors gravity and day, and spider ID as a random factor. The interaction was omitted because its *p* value was > 0.8.

### Other aspects

We defined the time of the beginning of web building to be the time of the last picture taken before the hub position was established (Zschokke and Vollrath 1995). Orb web nomenclature follows (Zschokke 1999).

## Results

### General observations

All spiders established themselves in their habitat and built their first web within 48 h of their release into the observation chamber. In general, webs were built around the time at which the lights were turned on and were removed around the time the lights were turned off 12 h later. In some cases, especially prior to moulting, webs were left intact for a few days. Web building followed the usual sequence of removing the previous web, exploration and building of primary radii and hub (which establishes the hub position, see above), followed by frame, radii and auxiliary spiral building, and finally the building of the sticky spiral (cf. Zschokke and Vollrath 1995). However, we never observed the species-typical renewal of two-thirds of the sticky spiral (Zschokke et al. 2006). All webs were either more or less round or, if they were asymmetric, the hub was near the edge of the web towards the top of the habitat; there were no webs, where the hub was near the edge in any other direction. Therefore, we considered only the vertical asymmetry for our analyses.

### Web asymmetry

**Fig. 3:**
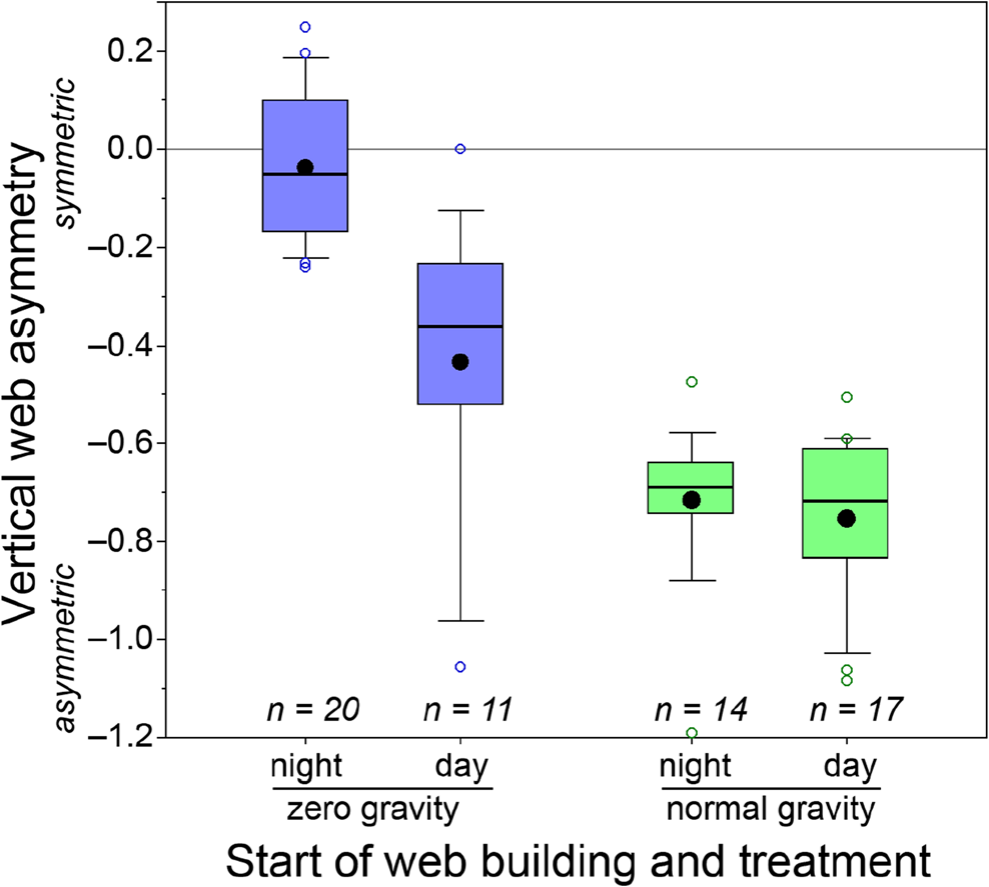
Vertical web asymmetry of *T. clavipes* webs built in zero gravity and normal gravity. Webs with an asymmetry of 0 have the hub in the centre of the web, whereas negative asymmetries denote hubs being above the centre. “Night” refers to webs whose building started *before* the lights were turned on; “day” refers to webs whose building started *after* the lights were turned on. Dots indicate average, whiskers indicate 95% percentiles and circles indicate outliers. All webs built in normal gravity were asymmetric—regardless when they were built—as is typical for this species. The asymmetry of webs built in zero gravity varied; webs whose building had started when the lights were off were all quite symmetric, whereas some webs whose building had started when the lights were on were asymmetric

The vertical asymmetry of webs built in zero gravity was quite variable (cf. Fig. 2a, b), but on average, they were vertically less asymmetric (*U* = 58.0, *p* < 0.0001; Fig. 3) than webs built in normal gravity, which all showed the species-typical vertical asymmetry (cf. Fig. 2d). Closer analysis revealed that all webs built in zero gravity, whose web building had started in the night, i.e. *before* the lights had been turned on, were quite symmetric, whereas *some* webs whose web building had started during the day, i.e. *after* the lights had been turned on, were quite asymmetric, similar to the webs built in normal gravity (*U* = 18.0, *p* = 0.0001). Webs built in normal gravity were all asymmetric, and there was no difference between webs whose web building had started in the night and those webs whose web building had started during the day (*U* = 97.0, *p* = 0.38). A mixed model analysis with individual as a random factor gave the same conclusions, indicating that there was no difference between the spiders.

### Spider orientation

**Fig. 4:**
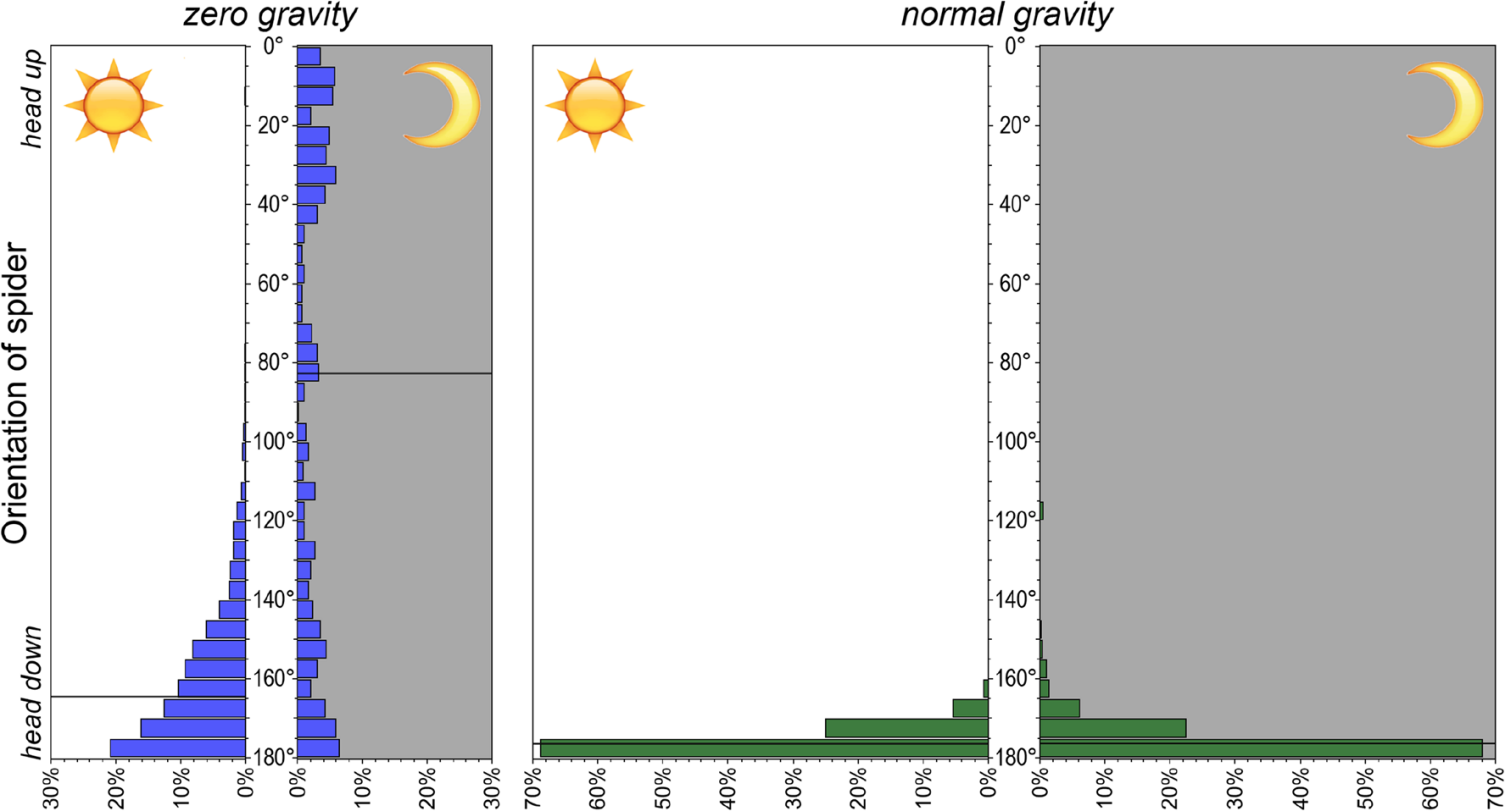
Frequency distribution of the spiders’ orientation whilst waiting on the hub. Shown are distributions under zero gravity and under normal gravity, as well as during the day (lights on) and during the night (lights off). Horizontal lines indicate the median. Sample sizes (number of pictures) were 6250, 456, 5395 and 1915, respectively. Spiders in normal gravity almost always faced downwards. Spiders in zero gravity tended to face downwards when the lights were on but showed a random orientation when the lights were off

**Fig. 5:**
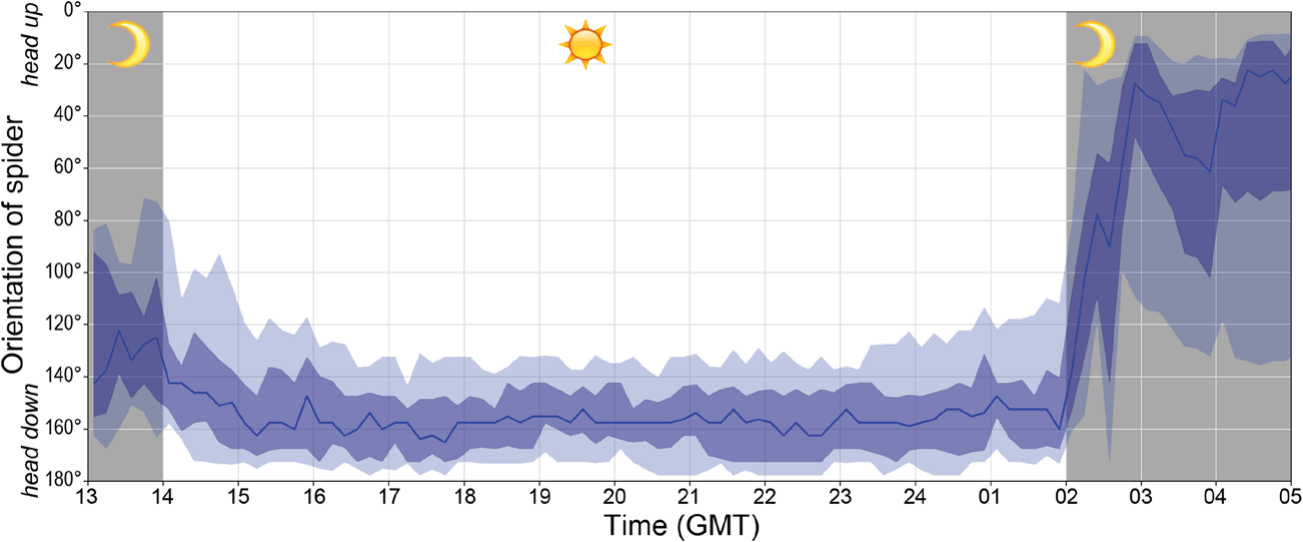
Change of orientation of one spider exposed to zero gravity in 32 webs over time of day (actual sample size per time slot varied, since some webs were only completed after the lights had been turned on and some webs were removed before the lights were turned off). The dark blue line indicates the median, the dark shaded area the 25 to 75 percentiles and the light shaded area the 10 to 90 percentiles. Whilst the spider was mainly facing downward (i.e. away from the light) during the time the lights were on, the orientation was quite random when the lights were off. Note the transition period of about 1 h after the lights had been turned on or off, respectively

The orientation of spiders waiting for prey on the hub of their web in zero gravity was quite variable, whereas spiders in normal gravity always oriented themselves downwards (median < 5° from vertically downwards, Fig. 4). Closer analysis revealed that spiders in zero gravity tended to orient themselves downwards when the lights were on (median = 15° from vertically downwards) but showed no tendency to face in any particular direction when the lights were off (median near horizontally; Fig. 4). It is noteworthy that the spiders retained their previous orientation for as much as 1 h after the lights had been turned on or off, respectively (Fig. 5).

These results strongly suggest that the spiders use the direction of light as a guiding cue when there is no gravity. To test our hypothesis that spiders orient themselves towards the larger part of the web when sitting on the hub, we could therefore consider the spider orientation only during the time the lights had been turned off. However, since we observed the spiders to change their orientation during the time the lights were off, we could not test our hypothesis.

### Web regularity

**Fig. 6:**
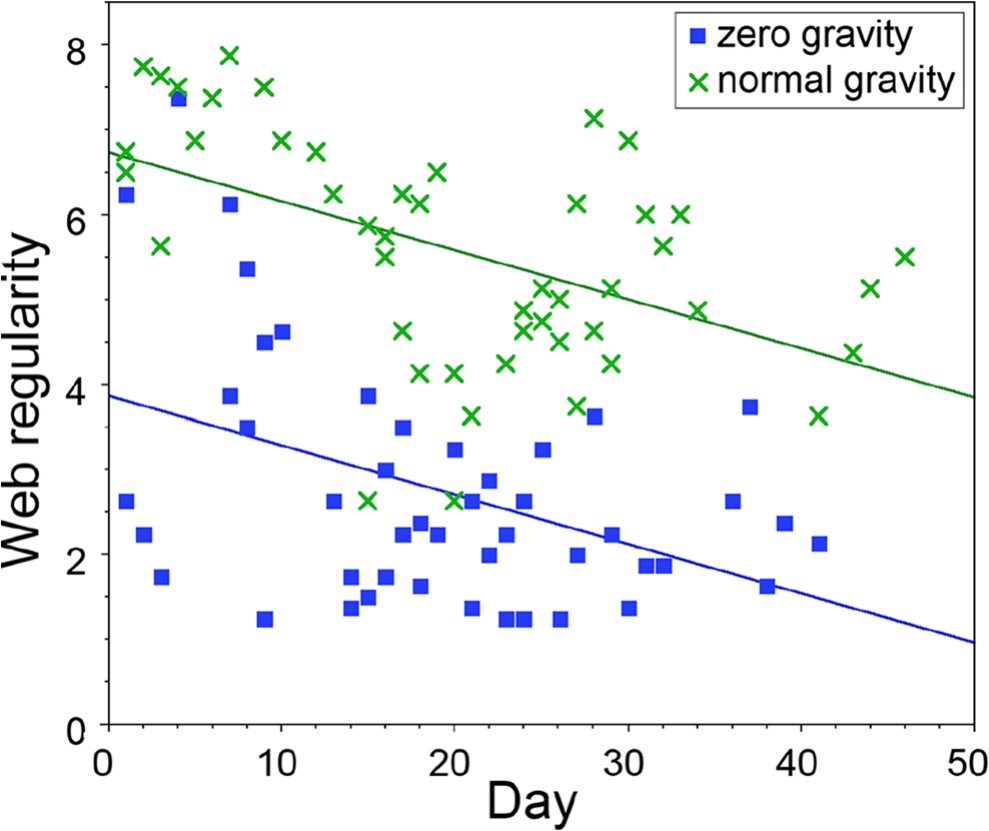
Decrease in the regularity of the *T. clavipes* webs built in zero gravity (squares) and normal gravity (crosses) during the course of the experiment

The average score for sticky spiral evenness was 2.18, for concentricity 1.55 and for gaps in the sticky spiral 0.43. Webs built in zero gravity had a lower regularity (average = 2.77, SD 1.41) than webs built in normal gravity (average = 5.56, SD = 1.34); in addition, web regularity decreased in similar ways for both zero gravity and normal gravity webs during the experiment (Fig. 6, Table 2).

**Table 2:** Summary of the analysis of the influence of gravity and day of experiment on web regularity. We used a general linearised mixed model (glmmPQL) with the factors gravity and day (without interaction) and spider ID as a random factor (*numDF*, degrees of freedom of numerator; *denDF*, degrees of freedom of denominator). We analysed 47 webs built in zero gravity and 47 webs built in normal gravity

Factor	numDF	denDF	*F* value	*p* value
Gravity	1	2	38.38	0.0251
Day	1	89	30.32	< 0.0001

### Other aspects

It is noteworthy, that both *T. clavipes* spiders set new records for web-building spiders. Spider A (a male) survived in zero gravity for 65 days and was still alive after it was returned to Earth. Spider B (a female) built a record number of 34 webs in zero gravity and moulted three times in zero gravity, demonstrating that spiders can repeatedly moult in zero gravity.

## Discussion

The results of our study only partially matched our expectations. Whilst most webs built in zero gravity were indeed much less asymmetric than the control webs built under normal gravity, some webs built in zero gravity still had a rather pronounced and consistent asymmetry; this was especially true for webs, whose building had started when the lights were on, suggesting that light replaced gravity as an orientation guide during web building. Since the web asymmetry is determined early during web building (Zschokke and Vollrath 1995), it was only relevant whether the light was on during that early stage of web building.

In addition, spiders in zero gravity showed a random orientation only during the time when the lights were turned off, whereas they quite consistently faced away from the lights, which were all placed along one side of the habitat, when the lights were on. This again suggests that light replaced gravity as an orientation guide when the spider was sitting on the hub of the web.

Since in normal gravity, and no matter whether the lights were on or not, spiders consistently built asymmetric webs and consistently faced downwards when sitting on the hub, we conclude that gravity is the most relevant orientation guide for spiders. Based on the observations of our experiments, we further conclude that the visual stimulus of the direction of light can serve as an orientation guide in the absence of gravity.

Visual stimuli have been considered to be largely irrelevant for web-building spiders, since they can build their webs and capture prey in complete darkness (Peters 1931; Foelix 2011). Nevertheless, it is not entirely surprising that visual stimuli can play a role in web-building spiders, since at least some web-building spiders use the position of the light to find their way back to the hub or retreat after prey capture. This has been shown for the horizontal way-finding in *A. diadematus* (which uses gravity as a cue for vertical orientation; Peters 1932; Crawford 1984) as well as for *Agelena labyrinthica* (Clerck) that builds horizontal sheet webs (Bartels 1929; Görner and Claas 1985). In addition, several orb-web-building spiders (including *T. clavipes*) have been reported to use ambient light as an important cue to start web building (Homann 1947; Le Guelte and Ramousse 1979; Eberhard 1990; Moore et al. 2016). This was also observed in our study, in which the spiders started web building in 67 of 106 cases (63%) within 1 h of the time the lights went on.

It may seem surprising that spiders, even though they and their ancestors had never experienced an environment without gravity, are nevertheless able to compensate this by using the direction of light for orientation. However, since there is always the possibility that the spider’s gravity receptors fail for some reason, having another sense to compensate for that is clearly beneficial. The gravity receptors of spiders have been suggested to be slit sensilla located between the prosoma and the opisthosoma, which can register the relative movements between these two body parts (Barth and Libera 1970). During web building, the relative position of these two body parts changes constantly, so it may be especially useful during web building to be able to use the direction of light as an additional sense to help with orientation.

We observed that webs built in zero gravity were generally less regular than those built in normal gravity and that the regularity of all webs decreased during the experiment. Since webs built in zero gravity were less regular than those built in normal gravity, we conclude that even though spiders were able to build webs in zero gravity, the lack of gravity did perturb web building to some degree. Interestingly, however, web regularity decreased during the experiment for both zero gravity and normal gravity webs to a similar degree, which suggests that the *decrease* in web regularity was not caused by the lack of gravity. Since we also observed a build-up of silk in the habitats during the experiment, we feel that it is likely that the laboratory conditions may have caused this decrease of regularity, since it is known that webs built in the laboratory differ from those built in nature (Sensenig et al. 2010). In nature, wind, rain and other animals remove unused silk. We suggest that the absence of these factors lead to the observed build-up of silk, which together with the lack of space (*Trichonephila* spiders usually build larger webs than the ones they could build in the habitats used in the present study) could be the reason for the observed decrease in web regularity over time.

## Data Availability

The data that support the findings of this study are available from the corresponding author, ZS, upon reasonable request.
